# Case report: Quantitative recognition of virtual human technology acceptance based on efficient deep neural network algorithm

**DOI:** 10.3389/fnbot.2022.1009093

**Published:** 2022-10-26

**Authors:** Xu Wang, Charles Chen

**Affiliations:** ^1^School of Government, Sun Yat-sen University, Guangzhou, China; ^2^Guangzhou Foreign Language School, Guangzhou, China

**Keywords:** digital virtual human, acceptance, deep learning, UTAUT model, neural network algorithm

## Abstract

With the advancement of artificial intelligence, robotics education has been a significant way to enhance students' digital competency. In turn, the willingness of teachers to embrace robotics education is related to the effectiveness of robotics education implementation and the sustainability of robotics education. Two hundred and sixty-nine teachers who participated in the “virtual human education in primary and secondary schools in Guangdong and Henan” and the questionnaire were used as the subjects of study. UTAUT model and its corresponding scale were modified by deep learning algorithms to investigate and analyze teachers' acceptance of robotics education in four dimensions: performance expectations, effort expectations, community influence and enabling conditions. Findings show that 53.68% of the teachers were progressively exposed to robotics education in the last three years, which is related to the context of the rise of robotics education in schooling in recent years, where contributing conditions have a direct and significant impact on teachers' acceptance of robotics education. The correlation coefficients between teacher performance expectations, effort expectations, community influence, and enabling conditions and acceptance were 0.290 (*p* = 0.000<0.001), −0.144 (*p* = 0.048<0.05), 0.396 (*p* = 0.000<0.001), and 0.422 (*p* = 0.000<0.001) respectively, indicating that these four core dimensions both had a significant effect on acceptance. Optimization comparison results of deep learning models show that mDAE and AmDAE provide a substantial reduction in training time compared to existing noise-reducing autoencoder models. It is shown that time-complexity of the deep neural network algorithm is positively related to the number of layers of the model.

## Introduction

The beginning of worldwide research on artificial intelligence (AI) traces back to the Dartmouth Symposium held in 1956, in which the American scholar McCarthy defined the concept of AI from an engineering perspective, and AI has accumulated enormous potential over the past 60 years. Education, as one of the important fields of AI applications, is moving toward a new ecology of AI education. In other words, AI is leading the transformation of education and becoming an essential factor in promoting the development of education information technology integration and innovation (Wenge, 2021; Kim and Shim, 2022). As the most revolutionary technology nowadays, it will be of great benefits in optimizing the teaching environment, intelligent assessment, personalized tutoring, identifying classroom deficiencies and enhancing the learning experience to facilitate accurate teaching. There is no doubt that this will shock the traditional education objectives, contents and processes. Therefore, along with the rapid development of the intelligent era, the education field will be facing greater challenges and should make full use of AI technology to deepen education reform comprehensively and build an intelligent, lifelong and personalized talent training system so that education can better serve and develop people (Woolf et al., 2013; Aoun, 2017; Ahmad et al., 2021).

With the new wave of AI development, practice and application of avatar education in the basic education stage is becoming increasingly popular and gradually becoming an important vehicle for the development of AI (Benitti, 2012). As a concrete manifestation of the change in the basic approach and methodology of teaching, it not only plays an important role in promoting students' innovative spirit, computational thinking, practical skills and social skills, but also facilitates the development of interesting learning courses and the construction of personalized (student-specific) learning environments. This is a key element in the effective practice and promotion of robotics education, with virtual teachers playing a pivotal role in delivering robotics courses and guiding students in robotics competitions. Currently, China's education and teaching model has transformed from the “teacher-centered” and then “student-centered” unilateral teaching to the current “dominant-subject” model. The dual-focused education and teaching model has evolved into the current “lead-subject” model. Only when teachers play their “leading role” well can students effectively play their “main effect.” The starting point for effective practice and application of robotics education in primary and secondary schools is the teacher, which requires not only careful planning of teaching activities and selection of appropriate media and technologies, but also the embedding of new concepts and ideas to compensate for the limitations of traditional teaching models and to expand the advantages of robotics education for teachers and students, thus effectively enhancing teaching effectiveness. Takuya Hashimoto, a Japanese scholar, introduced a self-developed robot teacher into elementary school science classroom teaching, where participating students could discuss relevant issues with the robot teacher, showing that robots have greater potential in elementary school science classroom teaching, not only to enhance learners' knowledge acquisition, but also to improve students' creativity and questions (Hashimoto et al., 2013). Russian academic Elena Ospennikova used a quasi-experimental approach to examine the possibilities of robotics education in science and mathematics curricula. The study selected 186 students from grades 7 to 9 as target subjects and over three years of experimental observation concluded that robotics is a key element in the multidisciplinary orientation of the teaching and learning process in schools (Ospennikova et al., 2015). Andri Ioannou introduced Nao, a humanoid robot developed by Aldebaran Robotics in France, to the education of children with autism (ASD), based on an in-depth analysis of the advantages of combining humanoid robot education with the development of social communication skills in children with autism. After a four-session intervention with a boy with ASD, the robot was found to be an effective way to promote independence and emotional expression in the education of children with ASD (Ioannou et al., 2015). Deep neural networks are an extremely popular research direction in artificial intelligence since 2012, as well as artificial intelligence algorithms for effective analysis and processing of big data (Wang et al., 2021). Its advantages include overcoming the disadvantages of time-consuming and labor-intensive manual feature design, more effective (exponential) distributed data learning by pre-training layer-by-layer data to obtain the primary features of each layer. Compared with shallow modeling approaches, deep modeling enables more detailed and efficient representation of actual complex nonlinear problems. This technique shows potential to efficiently solve quantitative recognition techniques (Foad et al., 2022; Wang et al., 2022).

Objectively, research on robotics education in China has accelerated in the early twenty-first century, however, the key to making robotics education truly effective lies in the ability of teachers to accept and use robotics-supported teaching models. Since teachers' understanding of the concept of informational teaching and learning of the implementation content are internal factors that limit the development of their informational teaching skills (Smith and Sivo, 2012). As a result, studying the acceptance of virtual (robot) education by primary and secondary school teachers as well as grasping its influencing factors are beneficial to the development of robotics education in primary and secondary schools. For this reason, based on the teachers' own perspective, this study draws on the integrated information technology acceptance model (UTAUT model) and the technology acceptance theory model, optimizes the construction of the teachers' acceptance model of robotics education based on deep learning algorithms, analyzes the influencing factors of primary and secondary school teachers' acceptance of virtual human education using the questionnaire method, as well as proposes corresponding countermeasures to provide reference for the effective implementation of robotics education at all levels of teaching.

## Models and research methods

### Deep learning-based construction of UTAUT model

#### UTAUT model

From the domestic and international studies on teacher IT acceptance models, it is found that UTAUT model is widespread in the field of IT acceptance research. However, by combing through the studies related to robotics education and teacher acceptance, finding that there are fewer studies exploring its effective promotion and implementation from the influence of teachers in the main body of robotics education. Therefore, based on the theoretical basis of the UTAUT model and characteristics of robotics teaching, this study explores the factors influencing teachers' acceptance of robotics education from the teachers' perspective.

**Figure 1:**
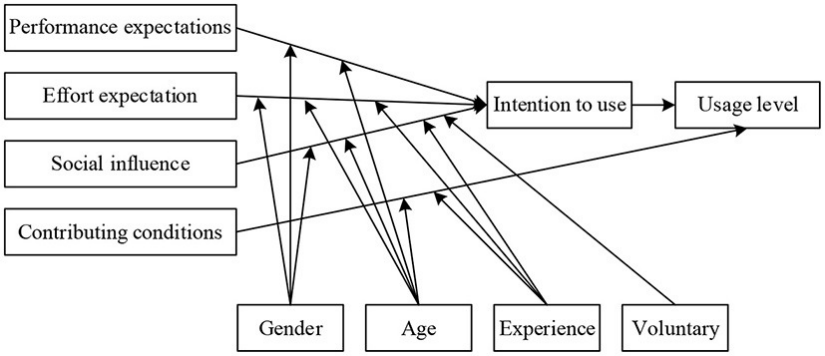
UTAUT model.

The UTAUT model was first proposed by Venkatesh et al. (2003). The model contains four core determinants of performance expectations, effort expectations, community influence and enabling conditions and four moderating variables of age, gender, experience, and voluntariness. As shown in Figure 1. This model explains 70% of technology adoption and usage behavior, outperforms previous technology acceptance models, which is now extensively applied to explore user acceptance behavior.

**Figure 2:**
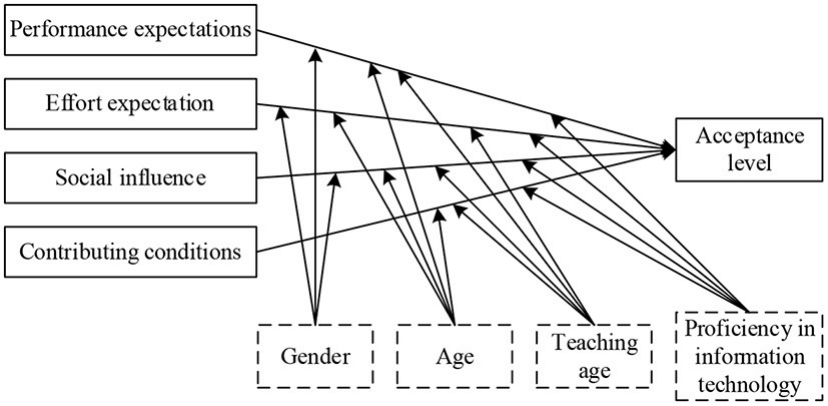
Theoretical model of factors influencing teachers' acceptance of robotics education.

To investigate the factors influencing teachers in carrying out robotics education, this study remained using the four core determinants in the UTAUT model. Since the development of robotics education in China is oriented to competition or club activities, both teachers and students have little access to robotics. Most teachers had little experience in using robotics and were not highly motivated to do so autonomously. As a result, two moderating variables, experience and voluntariness, were removed, while teaching experience and IT proficiency were added as moderating variables in conjunction with the technical characteristics of robotics education in primary and secondary schools and expert interviews. In addition, considering that acceptance includes both individual's own behavior and individual's attitude toward the object, both usage intention and behavior in the original model are collectively referred to as acceptance level, A theoretical model of factors influencing the acceptance of teacher robotics education is proposed, as shown in Figure 2.

#### Improvising approach based on deep learning

Deep learning network model involves inputting the original input data into a neural network containing multiple implicit layers, through nonlinear operations in the middle multiple implicit layers, where final output of the implicit layers is the deeper, abstract depth features learned from the input data through this deep network model (Guan et al., 2020). However, certain datasets without initial labels to whether the initial labels are involved in the whole network training process will be divided into three categories of deep feature learning, namely supervised feature learning, semi-supervised feature learning and unsupervised feature learning, where supervised feature learning of which can also be referred to as classification, semi-supervised feature learning between the two, which refers to the presence of both labeled and unlabeled data in the trained data, unsupervised supervised feature learning is also known as clustering (Gu et al., 2014).

The Expectation Maximization (EM) algorithm was first proposed by Dempster et al. The EM algorithm has a wide range of applications. The EM algorithm is utilized in numerous algorithms in machine learning (Intisar and Watanobe, 2018; Goulden et al., 2019). Such as the K-means, Support Vector Machine (SVM) (Ukil, 2007), GMM, Hidden Markov Mode (HMM) (Arica and Vural, 2000), Topic Generation Model LDA (Latent Dirichlet Allocation) (Hoffman et al., 2010), as well as various other models in which parameter estimation EM algorithm is used. It refers to solving some target parameters from the entire data set including hidden variables by iterative iterations employing a strategy of great likelihood estimation. Iteration of the EM algorithm is done by two main steps, E-step (Expectation Step) and M-step (Maximization Step). The expectation of each step of the expectation maximization algorithm is to calculate expectation of the model based on the hidden state of the model, after which Gaussian distribution of the conjectured hidden data is calculated, then fixed model parameters using maximum likelihood estimation to calculate the complete result containing both observed and hidden data, followed by the execution of M-step to finally obtain the parameters of the Gaussian mixture model. The E- and M-step are iterated until the parameters of solved Gaussian mixture model are approximately unchanged. Algorithm convergence is achieved and optimal expectation, covariance matrix and weights of each Gaussian distribution are obtained for the Gaussian mixture model. Expectation of the log-likelihood function of the mixture model is illustrated by the initial values of model parameters that have been selected, as defined in Equation (1):


(1) $$\begin{array}{l} E_{Q}\left[\log p\left(\theta |Y,Q\right)|\theta^{\left(i\right)},Y\right]=\int \log\left[p\left(\theta |Y,Q\right)\right]p\left(Q|\theta^{\left(i\right)},Y\right)dQ \end{array}$$

where, *Q* denotes implicit data that fail to be observed, θ^(i)^ denotes posterior standard deviation after the *i*+*1*st iteration. Conditional expectation probabilities of the joint distribution of the hybrid model can be expressed by Equation (2) as follows:


(2) $$L\left(\theta ,\theta_{i}\right)=\sum_{\;}^{m}\sum P\left(z_{i}|x_{i},\theta_{j}\right)\log P\left(x_{i},z_{i}|\theta \right)$$

Extreme values of the parameters of the log-likelihood function with conditional probabilities can be bounded by Equation (3) as follows:


(3) $$\begin{array}{l} \theta^{j+1}=\arg\max_{\theta }L\left(\theta ,\theta^{j}\right) \end{array}$$

The above E- and M-step are iterated continuously, terminating the iteration when θ^(i)^ and θ^(i+1)^ are infinitely close to each other.

### Theoretical model optimization of technology acceptance based on Elman neural network

#### Elman neural network model

Compared with the ordinary neural network structure, a new takeover layer is incorporated in the Elman network, where the implicit layer transmits processed data to the takeover layer, which memorizes incoming information from the implicit layer and uses received data together with the input layer input at the next moment as the input to the implicit layer at the next moment (Cheng et al., 2002). By storing it through the takeover layer and outputting it to the hidden layer at the next moment, it makes neural network have dynamic memory recognition of historical input data and enhances its ability to treat dynamic information. Its specific mathematical model is:


(4) $$\{\begin{array}{l} h(k)=g\left(w_{3}\cdot q(k)\right)\\ q(k)=f\left[w_{1}\cdot q_{c}(k)+w_{2}(u\cdot (k-1))\right]\\ q_{c}(k)=q(k-1) \end{array}$$

where, *h* represents output of the output layer, *g*() represents transfer function of the output layer, *w*_3_ represents weight of data received by the output layer that is processed by the implicit layer, *q* represents state of the implicit layer, and *k* represents current moment. In the second equation, *f* represents processing function of the implicit layer, Sigmoid is chosen in most cases, *w*_1_ represents weight of data processed by the implicit layer in the total received data of the takeover layer, *w*_2_ represents weight of the information received by the input layer transmitted to the implicit layer, *u* represents input of the input layer; *q*_*c*_ in the second and third equations refers to the state output for the takeover layer, and *k-1* in *q* indicates the previous moment.

#### Diffusion of innovation theory

Diffusion of Innovation Theory (DIT) was first introduced in 1962 by Everett M. Rogers, an American scholar, who used certain channels to make members of a social group more open to adopting new concepts and things. It emphasizes that an innovation is a thought or concept that can be perceived as novel by an individual or a social community. Diffusion of innovation is the process by which a new product spreads through a social system over a period of time through appropriate communication channels. DIT is divided into five groups of adopters based on the sequence of adoption and usage of innovations: (1) Innovation pioneers: first to adopt and use innovations with a spirit of discovery, accounting for 2.5% of the total; (2) Early adopters: highly visible, adopting and using innovations after the innovation pioneers, accounting for 13.5% of the total; (3) Early adopters: those who take longer to adopt and use innovations with more deliberation than innovation pioneers and early adopters, 34% of the total; (4) Late adopters: those who accept decisions only when they are clearly guided by the norms in the social system, 34% of the total; (5) Conservatives: those who are the last in the social network system to adopt and use innovations, 16 % of the total.

#### Theory of rational behavior

**Figure 3:**
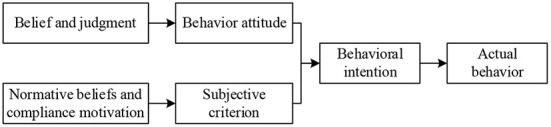
TRA model.

Theory of Rational Behavior (TRA) was co-proposed by American scholars Fishbein and Ajzen in the 1970s to explore the correlation between an individual's internal attitude toward a behavior and the actual performance of that behavior. TRA model has its origins in psychology and covers three basic assumptions: first, social groups are rational and able to accept and utilize knowledge and experience they acquire based on a systemic and holistic view; second, unconscious latent variables do not influence actual behaviors of social groups; and third, individuals themselves entirely determine their own conscious behaviors. The TRA model is given in Figure 3, from which it can be noticed that behavioral intentions in the TRA model can effectively infer actual behaviors used by individuals; while individual attitude and subjective norm that an individual displays when performing a certain behavior can effectively infer one's behavioral intention.

#### Theory of planned behavior

**Figure 4:**
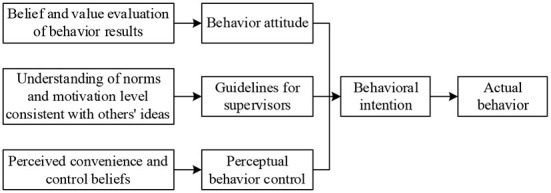
TPB model.

Theory of Planned Behavior (TPB) was first proposed by American psychologist Ajzen in 1985 to compensate for the limitations of the TRA model (Mahlaole, 2021). TPB model is considered as an extension and improvement of the TRA model, which can make fuller predictions and more convincing explanations of human behavior, as presented in Figure 4. The discrepancy between TPB and TRA lies in the predictors of individual behavioral intentions. In addition to subjective norms and attitudes, which are included in the TRA model, TPB also adds potential variable of perceived behavioral control (PBC). It refers to the perceived ease of performing a behavior. When individuals perceive that they have more opportunities and resources, their internal expectation of behavioral control increases, while the perceived constraints are reduced.

#### PSD learning algorithm

By analogy with the Widrow-Hoff (WH) learning rule the following equation can be obtained (Hinton and Nowlan, 1990):


(5) $$\begin{array}{l} \Delta w_{i}=\alpha x_{i}\left(y_{d}-y_{o}\right) \end{array}$$

where, *w*_*i*_ denotes weight of the *i*th input counterpart, α denotes learning rate, *y*_*d*_ denotes desired sequence, *y*_0_ denotes the actual output sequence, and *x*_*i*_ denotes sequence of inputs. Since the actual output is a sequence containing pulse spikes, it is challenging for derivation, and a derivable continuous value is obtained by convolving a sharp pulse with a convolution kernel when the PSD rule, defining as:


(6) $$\begin{array}{l} K\left(t-t^{j}\right)=V_{0}\cdot \left(\exp\left(\frac{-\left(t-t^{j}\right)}{\tau_{s}}\right)-\exp\left(\frac{-\left(t-t^{j}\right)}{\tau_{f}}\right)\right) \end{array}$$

### Research methodology

#### Questionnaire design

In this study, based on the relevant mature scales from existing studies, we designed independently measurement items for each variable in the context of the real situation of robotics education in less developed regions. In order to ensure the reliability and validity of questionnaires, author conducted two rounds of research. In the first round, 35 robotics teachers were randomly selected, followed by a revision of the questionnaire based on the initial research results to better match the real situation of robotics teachers in less developed regions. Final developed formal questionnaire consisted of the following two components with 33 question items. The first part is a survey of basic information of primary and secondary school teachers, with 15 question items, including gender, teaching age, title, school nature, school location, proficiency in information technology, frequency and barriers to robotics education, etc. The second part is a survey of factors affecting teachers' acceptance of robotics education, including five dimensions, namely, performance expectancy (PE), effort expectancy (EE), community influence (SI), enabling conditions (FC), and acceptance (AD), with a total of 18 questions. To ensure robotics teachers' recognition of the questionnaire answers, these measurement questions were in the form of a five-point Likert scale, with 1–5 indicating strongly disagree, disagree, neutral, agree, and strongly agree, respectively.

#### Questionnaire reliability and validity analysis

Reliability (reliability) focuses on the accuracy, consistency and stability of the recovered sample data. That is, the magnitude of the variability of the measurement results by the random errors generated during the measurement process. Before the formal questionnaire is distributed, reliability testing is normally conducted to purify the content of the questionnaire. The value indicating reliability index is called reliability coefficient, which is correlation coefficient between the results obtained by two or more tests, mostly distributed in the range of 0–1. Reliability tests mainly include test-retest reliability and internal reliability, in which a scale is repeatedly tested on the same target object at different times and the degree of similarity of the test results is then determined. However, repeated tests possibly have the following problems: first, there will be variability in the measurement subject's own cognitive level after having one subject experience; second, the measurement subject may change somewhat when a subject is measured twice or more. Therefore, most experts and scholars use internal reliability to calculate reliability size. Metrics for detecting internal reliability are generally θ coefficient, Ω coefficient, Cronbach Alpha confidence coefficient (Cronbach's α), and total correlation coefficient of calibration items (CITC). Among them, CITC and Cronbach's α are the more commonly used methods for reliability evaluation.

- Cronbach's α reliability analysis

Cronbach's alpha captures both degrees of internal consistency and correlation among test items and is defined in Equation (7).


(7) $$\begin{array}{l} \alpha =\frac{\text{K}}{\text{K}-1}\left(1-\frac{\sum \overset{2}{\underset{i}{S}}}{S^{2}}\right) \end{array}$$

where, α represents Cronbach's alpha coefficient, K represents total number of questionnaire items, $S_{i}^{2}$ represents variance corresponding to the *i*th measurement item, and *S*^2^ represents variance of the whole questionnaire item scores.

When a measurement questionnaire involves several unrelated contents (i.e., different dimensions), it is required to test the internal reliability corresponding to each dimension separately, and on this basis to calculate the internal reliability of the whole questionnaire, instead of directly calculating the alpha coefficient 1 of the whole questionnaire. the reason for this is primarily because questions under the same dimension all reflect the characteristics of a certain aspect and have a high correlation, while the whole questionnaire needs to examine the comprehensive consideration of a certain “coverage,” thus there are differences between the one and the other. Larger Cronbach's alpha values indicate better correlation among the items. In general, Cronbach's alpha values >0.9 indicate excellent reliability, as well as Cronbach's alpha values between 0.7 and 0.9 indicate good reliability, meaning that the questionnaire scale is still acceptable. However, if Cronbach's alpha value of each measurement dimension (subscale) is <0.6 and Cronbach's alpha value of the total scale is <0.7, it is determined that internal consistency of the scale is inferior and questionnaire needs to be redesigned. Based on the summary of several researchers' views on Cronbach's alpha value, Ming-Lung Wu divided reliability value testing criteria in detail, as shown in Table 1.

**Table 1:** Cronbach alpha test criteria.

**Cronbach's alpha value**	**Credibility**
Cronbachα≥0.9	Extremely credible
0.7≤Cronbachα <0.9	Credible (more common)
0.5≤Cronbachα <0.7	Credible (most common)
0.4≤Cronbachα <0.5	Credible
0.3≤Cronbachα <0.4	Less credible
Cronbachα <0.3	Not credible, should be deleted

- Total item statistics analysis

CITC is designed to measure correlation coefficients between each item and its dimension, in order to remove “junk” items from the questionnaire and clean up the content. There is no unanimous opinion on the evaluation criteria of CITC. Foreign scholar Cronbach considered that questions with CITC <0.5 should be discarded, while domestic scholar Lu Zhendai considered that questions with CITC >0.3 should be retained. The criteria for eliminating items in this study were based on two principles proposed by Cronbach: first, CITC is <0.5; second, alpha coefficient of the deleted item exceeds alpha coefficient of the variable to which it belongs, i.e., the reliability of the potential variable corresponding to the item has improved significantly. Results are shown in Table 2, and items were removed when they met both of these principles. To ensure the scientific validity of the study, two rounds of research were conducted with teachers participating in the “Virtual Human Education in Guangdong and Henan Primary and Secondary Schools” as the research subjects. Thirty-five teachers were selected for the first round of research, formal research was conducted by online questionnaire, 203 questionnaires were collected, of which 190 were valid. The survey results showed that 64.21% of the teachers who participated in robotics education training were male teachers; 78.42% of the teachers were aged between 26 and 45; 96.84% of the teachers' education was concentrated in college and undergraduate level, and only 2.11% of the teachers were graduate and above; 72.11% of the teachers' teaching experience was concentrated in 6–15 years, 15 years or above; 68.42% of the teachers' titles were concentrated in secondary school level 2 and secondary school level 1. In terms of the surveyed teachers' proficiency in IT, 61.58% were competent; teachers from public schools accounted for 98.95%, while teachers from private schools accounted for only 1.05%; rural teachers accounted for 38.95%, while urban teachers accounted for 61.05%. In addition, in terms of the school year in which the teachers serve the students, due to the shortage of teachers in robotics education in Henan Province, there is still a phenomenon that the same teacher teaches students in different grades; therefore, total number of teaching grades involved in the surveyed teachers is >190, covering 67.89, 34.21, and 11.05% of elementary, middle, and high schools, respectively.

**Table 2:** Results of exploratory factor analysis.

**Sample**	**Ingredients**				
	**1**	**2**	**3**	**4**	**5**
EE1	−0.145	0.654	0.073	−0.012	0.032
EE2	−0.022	0.779	−0.038	0.123	−0.024
EE3	−0.081	0.678	−0.125	−0.042	0.386
EE4	−0.076	0.687	0.168	0.028	−0.298
EE5	0.089	0.668	0.036	−0.125	−0.325
PE1	0.037	0.038	0.134	0.884	0.039
PE2	0.020	−0.006	0.084	0.788	0.062
PE3	0.373	−0.002	−0.025	0.587	0.042
FC1	0.168	−0.098	0.305	0.101	0.808
FC2	0.312	−0.081	0.202	0.045	0.798
S11	0.219	0.068	0.827	0.085	0.152
S12	0.102	0.018	0.878	0.067	0.152
S13	0.219	0.041	0.698	0.192	0.119
AD1	0.702	−0.205	0.258	0.021	0.142
AD2	0.788	−0.198	0.231	0.078	0.143
AD3	0.888	−0.087	0.064	0.010	0.095
AD4	0.878	0.015	0.119	0.052	0.087
AD5	0.787	0.095	0.079	0.118	0.058

## Research results and analysis

### Relationship analysis of teachers' acceptance of robotics education and influencing factors

Based on the results obtained from the sample data processing analysis to validate initial model and research hypotheses, this study revealed that effort expectation, perceived enjoyment, and performance expectation were factors that directly influenced teachers' acceptance of robotics education, while enabling conditions, community influence, and innovation expectation significantly and indirectly influenced acceptance, and perceived enjoyment could also indirectly influence acceptance through community influence, which will be analyzed and discussed in detail next.

#### Effect of performance expectations and effort expectations on acceptance

Performance expectations and effort expectations in robotics education positively and directly affect teachers' acceptance (Hl, H2), i.e., the higher performance expectations (PE) or effort expectations (EE) that teachers place on robotics education, the stronger their acceptance of robotics education. This conclusion is not only consistent with the original UTAUT model, but also with earlier research findings (Almaiah et al., 2019; Raffaghelli et al., 2022). Analysis of the paths revealed that performance expectations (path coefficient of 0.117) had a slightly stronger effect on acceptance than effort expectations (path coefficient of 0.101), generally speaking, teachers' willingness to attempt to introduce a new teaching model into their actual classrooms will heavily consider whether the model contributes to their performance levels. For virtual human teachers, if the implementation of robotics education causes them to feel a shift in their role and facilitates their salary, title, or promotion opportunities, which truly leads to more professional development, then this will undoubtedly strengthen their belief in practicing and applying robotics education. Descriptive statistical analysis of the core variables showed that teachers scored higher on the performance expectation level for questions PE-1 and PE-4, with scores of 3.73 and 3.68, respectively, indicating that most teachers perceived robotics education as both a key component in transforming their teaching role and an ideal platform for their professional growth, which facilitated their acceptance of robotics education. However, scores for PE-2 and PE-3 were low, at 3.38 and 3.10, respectively, which indicates that teachers in the current regional basic education level basically do not receive additional rewards for carrying out robotics education, and to some extent, it may also weaken teachers' enthusiasm to carry out robotics education. Therefore, in the process of promoting the practical application of robotics education, teachers' awareness of the concept of robotics education needs to be strengthened. In the process of actively organizing training in robotics project instruction and teaching skills, attention needs to be paid to the role of robotics education in teachers' professional development and to improving relevant assessment and reward mechanisms.

#### Effects of innovation expectations and facilitating conditions on effort expectations

Enabling conditions and innovation expectations in robotics education positively influenced virtual teachers' own effort expectations (H7, H8), i.e., more innovative teachers or more adequate accommodations already in place, the greater teachers' effort expectations, which enhanced their acceptance of robotics education. This is consistent with earlier research findings as well. The path analysis indicated that innovation expectations (path coefficient of 0.329) acted slightly more on effort expectations than enabling conditions (path coefficient of 0.294). Innovation expectations refer to the degree of teachers' innovativeness and problem-solving intentions when confronted with a new technology or a new pedagogical paradigm, which contributes to teachers' beliefs about the acceptance of a new technology or a new pedagogical paradigm (Rosenbusch et al., 2019). In general, if teachers frequently follow the latest developments of emerging technologies and are particularly willing to experiment with the introduction of new educational ideas into the actual classroom when they are exposed to them, they may not deplete excessive time to pay attention to whether such teaching ideas will affect the teaching order and their own emotions, but whether they are able to understand it or apply it or encounter obstacles to overcome it better as soon as possible, and such teachers are relatively more confident in accepting new teachers are relatively confident in accepting new technologies or teaching ideas, their willingness to try new teaching models is largely to satisfy their curiosity. Conversely, if teachers are reluctant to introduce new teaching models into the classroom, they may subconsciously believe that implementing models will make the classroom disorderly and stressful in guiding students in the process of project practice, which in turn will increase their teaching tasks. Therefore, teachers should be trained to be creative and innovative at the level of their subjectivity and practical activities in receiving robotics education.

#### Effects of perceived pleasantness and innovation expectations

There is a positive direct effect of teachers' perceived pleasantness on robotics education on their level of acceptance (H6), i.e., the stronger teachers' perceived pleasantness on robotics education, the stronger their internal level of acceptance, which is consistent with earlier findings (Adieze, 2016). By comparing path coefficient values for each factor, it is observed that the direct effect of perceived pleasantness on acceptance is as high as 0.852 (*p* = 0.000 < 0.05), which is significantly higher than the effect of each other variable. One possible reason for this is that virtual teachers have a strong interest in novelty or new teaching models, they want to be more enjoyable and stimulate their curiosity and exploration, rather than teaching in the traditional test-based education model for a long time, whereas robotics education as an existing new teaching concept can largely lead them to explore new knowledge and stimulate their curiosity, which makes it prone to feel enjoyable and enhance their motivation and interest in teaching, which in turn significantly enhances their belief that they tend to accept the model. As indicated by the descriptive statistical analysis of core variables, teachers' scores on the question items PP-1, PP-2, and PP-3 in the perceived pleasantness dimension were roughly comparable, with scores of 3.80, 3.82, and 3.75 respectively, indicating that most teachers have favorable perceived pleasantness of robotics education and are willing to actively attempt robotics education, however, they are still between neutral and agree (mean value of 3.79 between 3 and 4). Therefore, in the process of promoting practical applications of robotics education, teachers' perceived enjoyment of robotics education can be further enhanced by training practical activities.

### Analysis of teacher acceptance of robotics education

#### Descriptive statistical analysis of questionnaires

Results of the study demonstrated that the scores for each variable ranged from 2.92 to 3.87, with the highest score for acceptance (3.87) and the lowest score for effort expectancy (2.92). The standard deviation of the variables is <1.0, which indicates that scores of the variables are densely distributed around mean values, and mean values are well-represented, as shown in Table 3.

**Table 3:** Descriptive statistical analysis of model variables.

**Variant**	**Average value**	**Standard deviation**	**Cronbach's α**	**Sum of Cronbach's α**
Performance Expectations	3.26	0.78	0.785	0.782
Effort Expectations	2.92	0.59	0.760	
Community Impact	3.39	0.85	0.808	
Enabling conditions	3.08	0.94	0.842	
Acceptance level	3.87	0.58	0.889	

#### Variance and regression analysis of teachers' acceptance of robotics education

Taking into account the different background characteristics of elementary and secondary school teachers, one-way ANOVA with independent samples *t*-test was employed in this study to explore the variability in the factors exhibited by teachers from different backgrounds. Results indicated that there were no significant differences in performance expectations, community influence, enabling conditions, and acceptance among teachers of different ages and titles, with significant differences only in effort expectations, suggesting that teachers with older ages and higher titles would perceive robotics instruction as more complex. There were significant differences in effort expectation and acceptance among teachers of different teaching ages, while teachers with more than 15 years of teaching experience showed a phenomenon of “low effort expectation and high acceptance,” indicating that teachers of higher teaching ages may perceive many barriers to robotics education, however, it is possible that they want to break through the limitations of the old teaching methods and are more receptive to new things. Teachers with different levels of IT acceptance reached significant differences in terms of effort expectations, enabling conditions, and acceptance levels. There were no significant differences between public and private teachers, urban and rural teachers on these five variables.

Relevance between variables was measured in this study by applying Pearson's product-difference correlation coefficient, and correlations basically existed between all dimensions (Um and Crompton, 1986). Among them, acceptance was positively correlated with performance expectancy, community influence and enabling conditions; since the questions about effort expectancy designed in this study were biased toward the reverse questions, as in the case of conducting robotics education that tends to create uncontrolled, stressful and time-consuming classrooms, results of negative correlation between effort expectancy and acceptance coincided with the design of this experiment; moreover, correlations between effort expectancy and acceptance were weak, results of which are shown in Table 4.

**Table 4:** Correlation coefficients of teachers' acceptance of robotics education and its various influencing factors.

**Dimension**	**Performance expectation**	**Effort expectations**	**Community impact**	**Enabling conditions**	**Acceptance level**
Performance expectations	1				
Effort expectations	0.003	1			
Community impact	0.308^***^	0.045	1		
Enabling conditions	0.232^***^	−0.169	0.418^***^	1	
Acceptance level	0.287^***^	−0.142^*^	0.397^***^	0.412^***^	1

* *p* < 0.05,

**p < 0.01,

*** *p* < 0.001.

**Figure 5:**
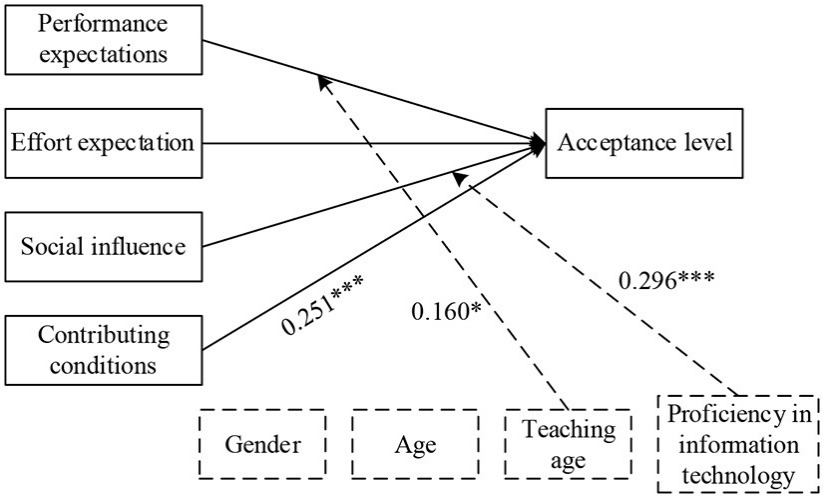
The path of influencing factors of virtual human teacher education acceptance.

To further validate the hypothesized model, multiple regression analysis was utilized in this study in an attempt to examine the causal relationships among the influencing factors. As seen in Table 4, the correlation coefficients between teacher performance expectations, effort expectations, community influence, enabling conditions, and acceptance were 0.290 (*p* = 0.000<0.001), −0.144 (*p* = 0.048<0.05), 0.396 (*p* = 0.000<0.001), and 0.422 (*p* = 0.000<0.001), respectively, indicating that that all four core dimensions had a significant effect on acceptance. In addition, previous one-way ANOVAs showed that moderating variables such as teachers' teaching experience and IT proficiency also had significant effects on acceptance, therefore, multiple regression analysis was attempted in this study to explore the specific effects of these variables on teachers' acceptance, and regression results are presented in Table 5. As seen in this, model 3 explained 28.2% of the results, while the adjusted R2 was finally chosen to explain 27.1% of the results, considering the sample size and the number of independent variables. In particular, enabling conditions had a significant correlation with acceptance; teaching age moderated effects of performance expectations on acceptance; community influence on acceptance was moderated by IT acceptance; effort expectations did not have a direct effect on acceptance of teacher robotics education. Through multiple regression analysis, a path diagram of factors influencing the acceptance of teacher robotics education can be obtained, as shown in Figure 5.

**Table 5:** Compound regression analysis of acceptance and coefficients.

**Model**	***R***	**Square *R***	**Adjusted *R***	**Square Std. Error of the Estimate**
1	0.432	0.196	0.191	2.689
2	0.506	0.254	0.250	2.587
3	0.530	0.281	0.270	2.655

### Effect analysis of neural network optimization

**Figure 6:**
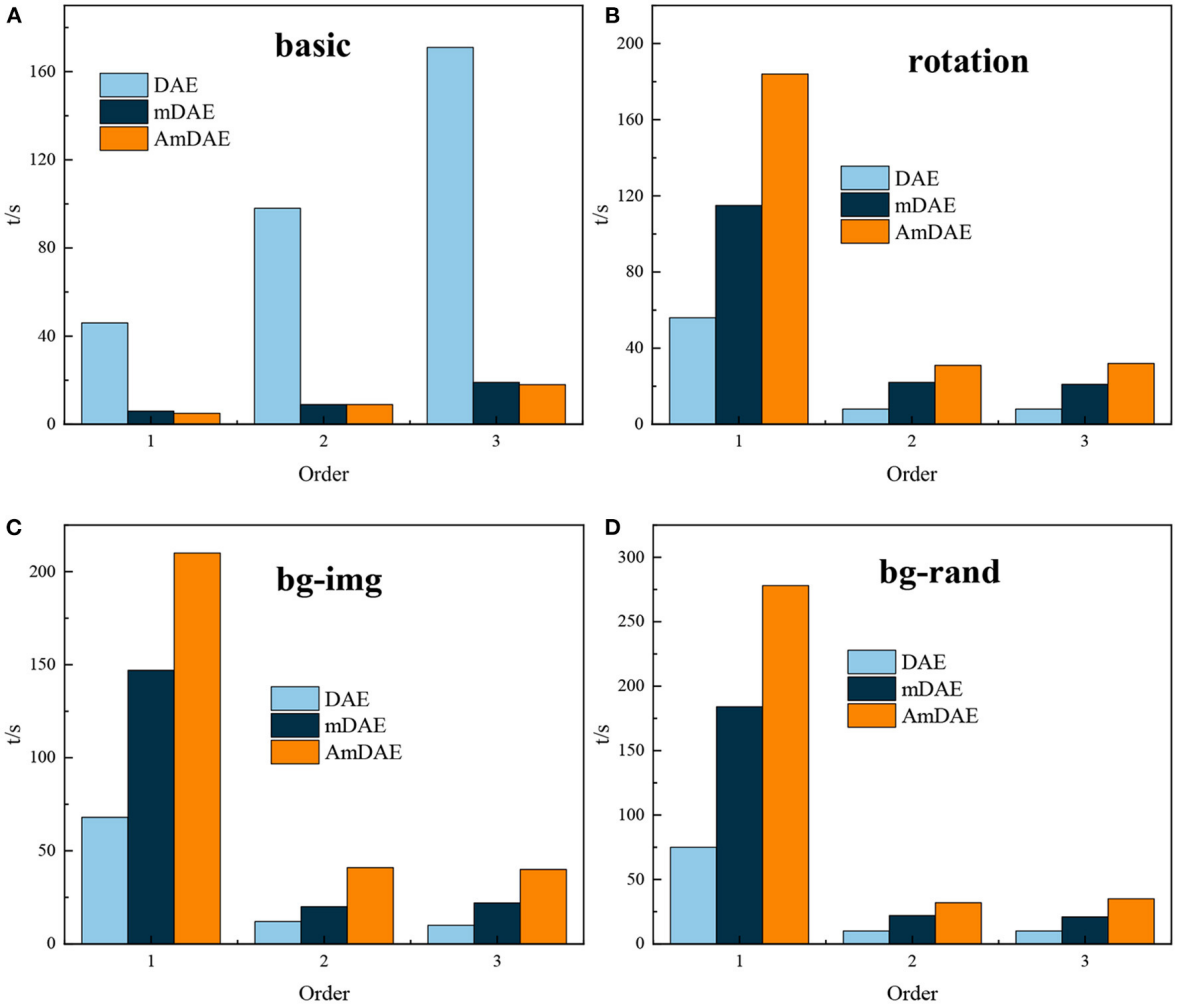
Time required for model training. **(A)** basic model, **(B)** rotating model, **(C)** background image, and **(D)** offset model.

To verify effectiveness of the proposed algorithms in this study. Existing deep neural networks composed of noise-reducing autoencoding (DAE), marginalized depth autoencoder (mDAE) and marginalized depth autoencoder with adaptive noise (AmDAE) were compared in experiments under the same conditions. Experiments were conducted to compare the algorithmic performance of the three algorithms, with statistics on the average time required to train the three deep neural network models once. Different implied layer building numbers of deep neural networks with different methods of model training time are shown in Figure 6.

As shown in Figure 6, mDAE and AmDAE have a substantial reduction in training time compared to the existing noise-reducing autoencoder model, reflecting the lower time complexity of the marginalization method, while the improved AmDAE and mDAE models take little difference in training time; model training time basically increases approximately linearly with the number of layers as the number of training layers increases for different standard MNIST variant datasets, indicating that the time complexity of the deep neural network algorithm is positively correlated with the number of layers of the model.

## Conclusion

Based on the UTAUT model, this study focuses on the main factors influencing the acceptance of virtual human education by teachers in order to promote application of robotics education in educational teaching activities, by taking some colleges and universities in Guangdong and Henan provinces as examples and draws the following basic conclusions.

- Robotics education is mostly taught by IT teachers, and there is a paradox of “low knowledge and high frequency.” Results of study showed that 53.68% of teachers were gradually introduced to robotics education in the last three years, which is related to the background of the rise of robotics education in school education in recent years.
- Descriptive statistical analysis, analysis of variance and reliability tests were conducted on the formal sample data using the appropriate software, while AMOS 22.0 software was used to test the correctness and rationality of the theoretical model and the 15 research hypotheses to obtain the final model that established the factors influencing the acceptance of robotics education by the virtual human teachers. Effects of influencing factors were in descending order: community influence < enabling conditions < effort expectations < performance expectations < innovation expectations < perceived pleasantness.
- Multiple regression analysis of model optimization based on neural networks showed that model 3 explained 28.2% of the results. Meanwhile, its explanatory ratio for the outcome reached 27.1%. In this case, enabling conditions have a significant correlation with acceptance; teaching age moderates effects of performance expectations on acceptance; community influence on acceptance is moderated by IT acceptance; and effort expectations do not directly affect acceptance of teacher robotics education.

## Data availability statement

The raw data supporting the conclusions of this article will be made available by the authors, without undue reservation.

## Ethics statement

The studies involving human participants were reviewed and approved by Sun Yat-sen University. The patients/participants provided their written informed consent to participate in this study.

## Author contributions

XW and CC put forward the core concepts and architecture of this manuscript. XW wrote this article. Both authors contributed to the article and approved the submitted version.

## Funding

This study was supported by Guangzhou Higher Education Innovation and Entrepreneurship Education Project (Creation based on Virtual Simulation and Artificial Intelligence New Entrepreneurship Curriculum and Teaching System Construction), Project No. 2019KC113.

## Conflict of interest

The authors declare that the research was conducted in the absence of any commercial or financial relationships that could be construed as a potential conflict of interest.

## Publisher's note

All claims expressed in this article are solely those of the authors and do not necessarily represent those of their affiliated organizations, or those of the publisher, the editors, and the reviewers. Any product that may be evaluated in this article, or claim that may be made by its manufacturer, is not guaranteed or endorsed by the publisher.
